# Characterization of Self-Cured Silicone Oils for Encapsulation of Ultraviolet-C Light-Emitting Diodes

**DOI:** 10.3390/polym17020250

**Published:** 2025-01-20

**Authors:** Xing Qiu, Qianhang Yu, Yuanjie Cheng, Jeffery C. C. Lo, Shi-wei Ricky Lee

**Affiliations:** 1 HKUST Shenzhen-Hong Kong Collaborative Innovation Research Institute, Futian, Shenzhen 518000, China; xqiuaa@connect.ust.hk (X.Q.); qyuam@connect.ust.hk (Q.Y.); 2 Smart Manufacturing Thrust, Hong Kong University of Science and Technology (Guangzhou), Nansha, Guangzhou 511400, China; 3 PKU-HKUST Shenzhen-Hongkong Institution, Nanshan, Shenzhen 518057, China; 4 Electronic Packaging Laboratory, Hong Kong University of Science and Technology, Hong Kong SAR, China; ychengao@connect.ust.hk (Y.C.); jefflo@ust.hk (J.C.C.L.); 5 Department of Mechanical and Aerospace Engineering, Hong Kong University of Science and Technology, Clear Water Bay, Kowloon, Hong Kong SAR, China; 6 Foshan Research Institute for Smart Manufacturing, Hong Kong University of Science and Technology, Hong Kong SAR, China; 7 LED-FPD Technology R&D Center, Hong Kong University of Science and Technology, Foshan 528200, China

**Keywords:** silicone oil, UVC LEDs, encapsulation

## Abstract

The effectiveness of ultraviolet-C light-emitting diodes (UVC LEDs) is currently limited by the lack of suitable encapsulation materials, restricting their use in sterilization, communication, and in vivo cancer tumor inhibition. This study evaluates various silicone oils for UVC LED encapsulation. A material aging experiment was conducted on CF1040 (octamethylcyclotetrasiloxane), HF2020 (methyl hydro polysiloxanes), and MF2020-1000 (polydimethylsiloxane) under UVC radiation for 1000 h. The analysis assessed transmittance changes and chemical composition alterations throughout the aging process. Notably, HF2020 showed an increase in transmittance before 500 h, indicating a curing process attributed to the photolysis of Si-H, leading to the formation of Si-O-Si. Further testing on 265 nm UVC LEDs, both with and without HF2020 encapsulation, showed that the encapsulated LEDs exhibited a remarkable maximum increase of 27% in radiant power compared to their unencapsulated counterparts. Additionally, these encapsulated LEDs sustained higher radiant power levels during the first 200 h of operation. Notably, its potential application in photodynamic therapy is significant; by activating photosensitizers with higher UVC exposure, it facilitates the rapid production of reactive oxygen species, leading to effective cancer cell destruction within a short timeframe.

## 1. Introduction

Ultraviolet-C light-emitting diodes (UVC LEDs) are employed in medical disinfection services, food safety [1], and confidential communication [2]. UVC LEDs, beyond their well-known roles in disinfection and communication, have shown promising potential in terms of inhibiting the growth and proliferation of cancer cells [3]. UVC LEDs have the ability to directly damage the DNA of cancer cells through mechanisms such as breaking, cross-linking, and mutating DNA strands when exposed to UVC light. This process not only disrupts the integrity of the DNA but also activates cellular pathways, including apoptosis, which inhibit the growth and proliferation of cancer cells [4,5,6,7]. Consequently, UVC LEDs demonstrate promising potential as an anti-cancer treatment. However, the limited radiant power of UVC LEDs hinders their disinfection and communication applications [8], and the lack of suitable encapsulation materials restricts their application in in vivo cancer tumor inhibition [9]. 

Enhancing the radiant power of UVC LEDs can be achieved through filling refractive index matching materials. A commonly used technique for UVC LED packaging involves a quartz lens [10]. However, a significant concern is the cavity between the LED chip and the lens, where UVC rays experience substantial total reflection losses as they transit between the LED chip and the surrounding air [11]. Researchers have explored filling this cavity with liquid silicone oil, specifically polydimethylsiloxane, to increase the radiant power of UVC LEDs. Polydimethylsiloxane maintains transparency for 200 h, and its liquid phase complicates the packaging process [11]. Another approach is the use of fluorine-containing resins to enhance radiant power, and the complex manufacturing process of -CF_3_ groups and the high costs are significant drawbacks [12].

The challenge in using UVC LEDs for in vivo cancer tumor inhibition is that traditional UVC LED devices are not implantable due to unsuitable encapsulation materials. High UVC transparent and biocompatible silicone oils are proposed as encapsulation materials for traditional UVC LED devices. Previous studies have shown that silicone oil offers high UVC transmittance and biocompatibility [11]. However, the polydimethylsiloxane cannot be cured, making it unsuitable as the encapsulation material for in vivo cancer tumor inhibition. 

Silicone oils, specifically polydimethylsiloxane (MF2010-1000), octamethylcyclotetrasiloxane (CF1040), and methyl hydrogenpolysiloxane (HF2020), have been investigated to determine their potential as encapsulation materials for UVC LEDs. Notably, this study presents the first exploration of octamethylcyclotetrasiloxane (CF1040) and methyl hydrogenpolysiloxane (HF2020) in the context of UVC LED encapsulation. Additionally, it marks the inaugural report on the curing characteristics of silicone oils when exposed to ultraviolet light for UVC LED applications. This research introduces self-curing silicone oils as innovative materials for UVC LED encapsulation, which effectively mitigates the radiant power loss issues commonly encountered with traditional UVC LED devices that utilize quartz lenses.

## 2. Materials and Methods

### 2.1. Materials and Samples Preparation

The silicone oils utilized in this study include MF2010-1000 (CH_3_[Si(CH_3_)_2_O]ₙSi(CH_3_)_3_), CF1040 ([(CH_3_)_2_SiO]_4_), and HF2020 ([CH_3_(H)SiO]ₙ), all of which are commercially available products supplied by SISIB Silicones, located in Nanjing, China. These products are well established and mass produced by the manufacturer.

The preparation of the UVC LED devices for aging tests involves the following steps: (1) obtain commercially available 3535 (3.5 mm × 3.5 mm) aluminum nitride ceramic carriers designed for UVC LEDs, along with UVC LED chips of model PCC-35-V2 from Photon Wave, Yonging, South Korea, and aluminum substrates; (2) employ Surface-Mounted Technology (SMT) to mount the UVC LED chips onto the ceramic carriers by the reflow process to form solder joints; (3) utilize a dispensing process to apply HF2020 into the cavity of the ceramic carrier containing the UVC LED chips; (4) cure the samples through UVC radiation, thereby completing the assembly of the UVC LED devices; (5) finally, mount the assembled UVC LED devices onto the aluminum substrates for subsequent aging tests.

### 2.2. Transmittance Characteriazation

**Figure 1:**
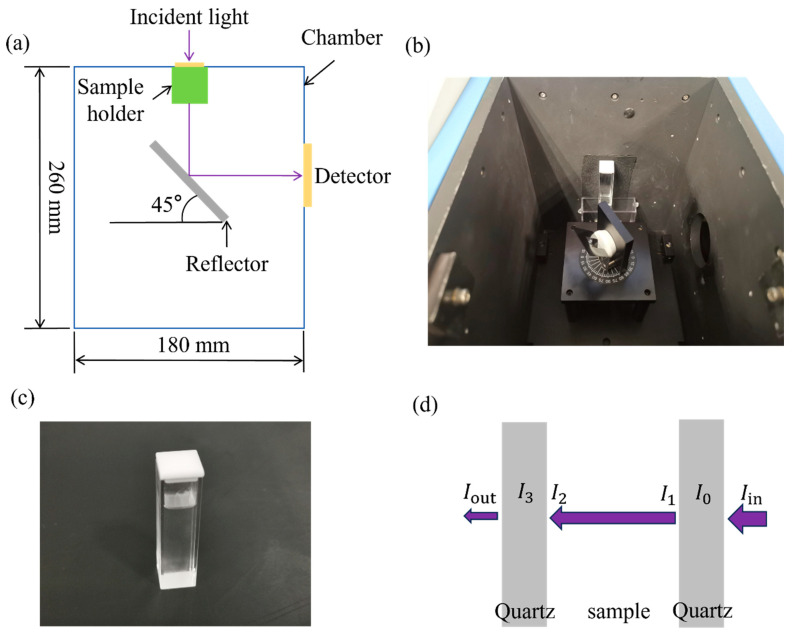
Transmittance test schematic diagram and setup: (**a**) schematic diagram; (**b**) setup; (**c**) quartz bottle with samples; (**d**) schematic diagram of the test situation.

In UVC LED packaging, the higher the transmittance of the filling material, the greater the radiant power emitted by the UVC LED. We determined the transmittance of these materials by assessing the intensity of light before and after passing through the filling substance. The transmittance of the test samples was evaluated using a fluorescence spectrometer (FluoroMax-4, HORIBA JOBIN YVON, Montpellier, France) at wavelengths of 265 nm and 280 nm, respectively, as illustrated in the experimental setup diagram in Figure 1a,b. In this setup, the incident light passed through a quartz container filled with the sample, was then reflected by a 45-degree angle reflector, and ultimately detected by a detector to measure the final intensity.

The intensity of light before and after passing through the quartz container can be quantified. However, when the sample was placed inside a quartz container, as depicted in Figure 1c, reflection occurred when light was transmitted from the air to the interior of the quartz material. As illustrated in Figure 1d, the path of the incident light was directed through the quartz container. To evaluate the transmittance of the specimen, it is necessary to measure the intensity of light passing through the sample inside the quartz container before and after. The detector was unable to record the incident light after passing through the specimen, as light was reflected when traversing the boundary between two materials with different refractive indices.

The Fresnel formula [13] was applied to calculate the intensity of the specimen before and after, so as to characterize the transmittance of the specimen. It was assumed that both the quartz container and the specimen in the experiment exhibited uniform properties and that the incident angle of the light was perpendicular. Assuming that the intensity of incident light is $I_{in}$, the intensity of light at the incident side of the quartz bottle is

(1) $$I_{0}=I_{in}\left[1-{\left(\frac{n_{1}-n_{2}}{n_{1}+n_{2}}\right)}^{2}\right],$$

where $n_{1}$ and $n_{2}$ are the refractive indices of air and quartz, respectively. Then, the light passed through the quartz layer and entered into the oil specimen. The intensity of the light after the light passes through the quartz layer is

(2) $$I_{1}=I_{0}\left[1-{\left(\frac{n_{2}-n_{3}}{n_{2}+n_{3}}\right)}^{2}\right],$$

where $n_{3}$ is the refractive index of the specimen. The light will be absorbed by oil. Before the light enters the quartz again, the intensity of the light is

(3) $$I_{2}=I_{1}T,$$

where T is the transmittance of the specimen. The intensity of the quartz closer to the detector side is

(4) $$I_{3}=I_{2}\left[1-{\left(\frac{n_{2}-n_{3}}{n_{2}+n_{3}}\right)}^{2}\right]$$

After the light passes through the quartz part and enters into the air, the intensity of the outcoming light is

(5) $$I_{out}=I_{3}\left[1-{\left(\frac{n_{1}-n_{2}}{n_{1}+n_{2}}\right)}^{2}\right].$$

The refractive indices of air, quartz, and oil are $n_{1}$ = 1_,_ $n_{2}$ = 1.46 [13] and $n_{3}$ = 1.4 [14], respectively. By detecting the intensity of incident light and outcoming light and combining Equations (1)–(5), the transmittance of the specimen, *T*, can be measured. For each specimen, we tested it three times and obtained the corresponding transmittance.

### 2.3. Material Aging Test and Results

**Figure 2:**
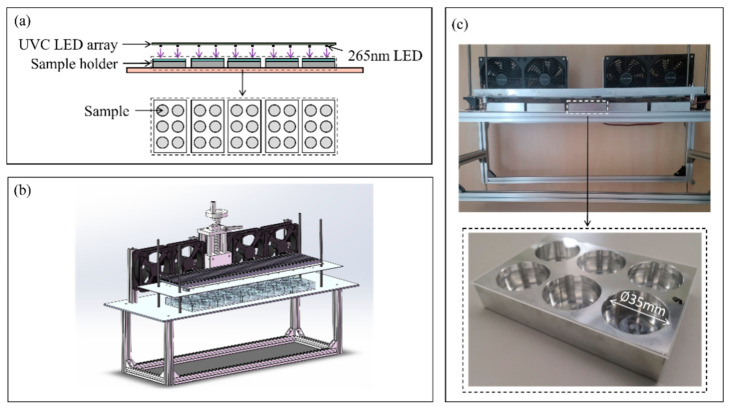
UVC aging test setup, (**a**) schematic diagram of aging test; (**b**) schematic diagram of the material aging setup; (**c**) experimental setup.

A specific piece of material aging equipment was designed for the UVC aging test. The configuration of this equipment is depicted in Figure 2a,b. Given that the LED package has an approximate divergence angle of 70 degrees, the specimens were placed at a distance of 10 mm from the LED chips to ensure uniform illumination of the entire surface of the silicone oil samples. To safeguard samples from contamination, a 1 mm thick quartz plate was placed over the sample holder. The completed experimental setup for the material aging test is illustrated in Figure 2c.

An irradiance meter (USR200, EVERFINE, Hangzhou, China) was utilized to assess the irradiance at a distance of 10 mm of each sample holder before subjecting them to the UVC aging test. The average irradiance of all sample holders was 33 W/m^2^, with a standard deviation of 1 W/m^2^. The irradiance characterization result demonstrated that the specially designed experimental setup can consistently deliver stable and uniform radiation to all 30 specimens.

**Figure 3:**
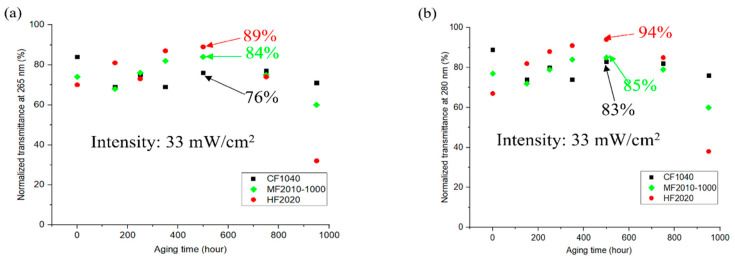
Transmittance at (**a**) 265 nm and (**b**) 280 nm with respect to aging time.

The irradiance was approximately 33 W/m^2^, and the transmittance test was conducted every 150 h during the aging test. Figure 3 shows the transmittance changes with the aging time. For CF, the transmittance gradually decreased with the increased aging time, although this decrease was less pronounced compared to MF and HF. The transmittance of CF can remain stable at around 70% during this 1000 h of aging time. For MF, the transmittance gradually increased from 70% to 85% during the first 500 h. However, the transmittance decreased rapidly in the second 500 h, from 85% to 60%. HF exhibited a similar trend with MF. The transmittance of HF gradually increased from 70% to over 90% in the first 500 h but then decreased sharply from 90% to below 40% by the end of the 1000 h aging period.

A viscosity test was conducted by a Hybrid Rheometer (Discovery HR-2, TA Waters, New Castle, DE, USA). The characterization temperature was set to 26 °C, the shear rate was 100 rpm/s, and the test duration was 180 s. Results are shown in Table 1. CF1040 did not show degradation, and its viscosity remained stable. The viscosity of MF increased with aging time and became solid after 750 h of aging. Changes in HF were faster than in MF. The viscosity of HF was initially lower than MF; however, after only 250 h of aging, it was higher than MF. The performance of HF underwent significant changes during the aging test. An amount of 265 nm radiation can solidify HF from a liquid state to a solid state.

**Figure 4:**
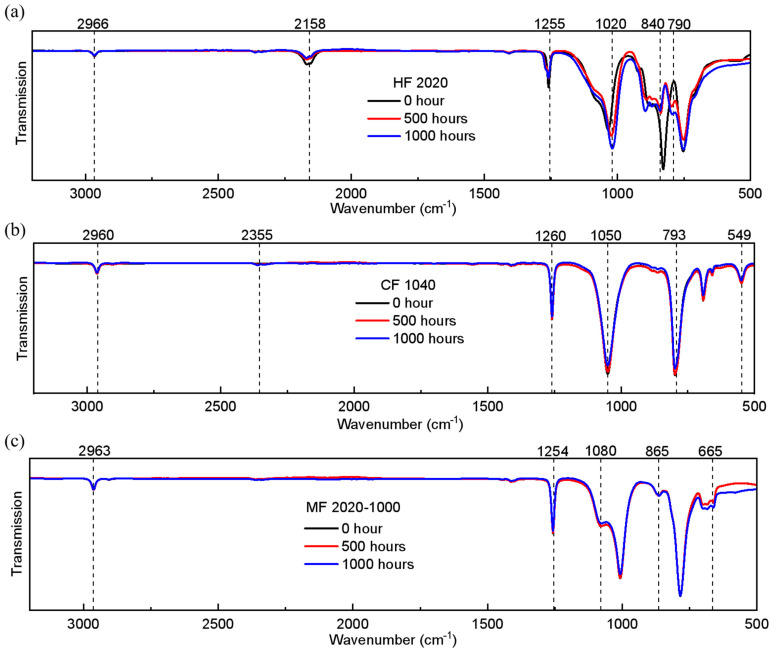
FTIR results (**a**) HF2020; (**b**) CF1040; (**c**) MF2020-1000.

HF2020 self-cured after 250 h of 265 nm irradiation, and the material state changed from liquid to solid, while CF1040 remained in the liquid state. The samples aged 0 h, 500 h, and 1000 h were tested by FTIR, and the results of the chemical group changes were characterized in Figure 4.

For HF, 2158 cm^−1^ is the characteristic absorption peak of the Si-H bond. The Si-H bond decreased gradually due to the photolysis of the Si-H bond under UV radiation. The Si-H bond gradually reduced and transformed into a -SiH_3_ free radical which was highly active towards the oxygen and easy to form into Si-O-Si. The stretching vibration of CH_3_-Si at 840 cm^−1^ gradually weakened, indicating an increase in the degree of cross-linking. The peak of 1020 cm^−1^ represented the vibration of the Si-O-Si skeleton. The peak domain gradually broadened as the aging time increased, indicating an increase in the Si-O-Si bond. In the beginning, the increase in Si-O-Si contributed to the transmittance of UV. However, after saturation of the cross-linking degree, the increase in the Si-O-Si bond became oversaturated after exceeding the saturation amount. This is the reason that the transmittance of UV increased in the first 500 h and then gradually decreased. 

For CF, there was a notable peak at 1260 cm^−1^, indicating the symmetrical bending vibration of the methyl group within the structure, while another peak at 793 cm^−1^ corresponded to the stretching vibration of CH_3_-Si. The chemical composition of CF demonstrated stability, aligning with its consistent macroscopic transmittance performance. The decline in peak at 1050 cm^−1^ was gradual and can be attributed to an increase in the cross-linking degree, leading to a decrease in transmittance.

In contrast, MF exhibited a peak at 1254 cm^−1^, indicating a symmetrical bending vibration in the methyl group within the structure. Peaks at 865 cm^−1^ and 784 cm^−1^ corresponded to the stretching vibration of CH_3_-Si. Another peak at 1080 cm^−1^ represented the characteristic Si-O-Si skeletal vibration, and its amplitude remains essentially unchanged. Overall, MF demonstrated consistent stability in its macroscopic characteristics.

The 265 nm UVC wavelength, characterized by its high energy, induces photolysis in materials, subsequently influencing the formation and cleavage of the silicone chemical bonds previously mentioned.

## 3. Results

Given that HF2020 exhibited the highest transmittance during the material aging test, a device aging test was conducted to evaluate the performance of the LED chip encapsulated with HF2020. The dispensed volume was determined by the radiant power, with a preference for higher radiant power. Table 2 illustrates the relationship between the UVC LED radiant power and the volume of HF2020. The UVC LED chip encapsulated with 4.0 μL of HF2020 achieved the highest radiant power, leading to the selection of 4.0 μL for the device aging test. The radiant power of a bare UVC LED chip was established as a standard working condition in the device aging test. Table 3 presents a comparison of the radiant power between the bare UVC LED chip and the UVC LED chip encapsulated with 4.0 μL of HF2020. The UVC LED chip encapsulated with 4.0 μL of HF2020 increased by 27% compared with the bare UVC LED chip in terms of radiant power. The working current of the UVC LED chip was 40 mA, and the radiant power of the tested UVC LEDs was characterized by the IS photoelectric test system.

**Figure 5:**
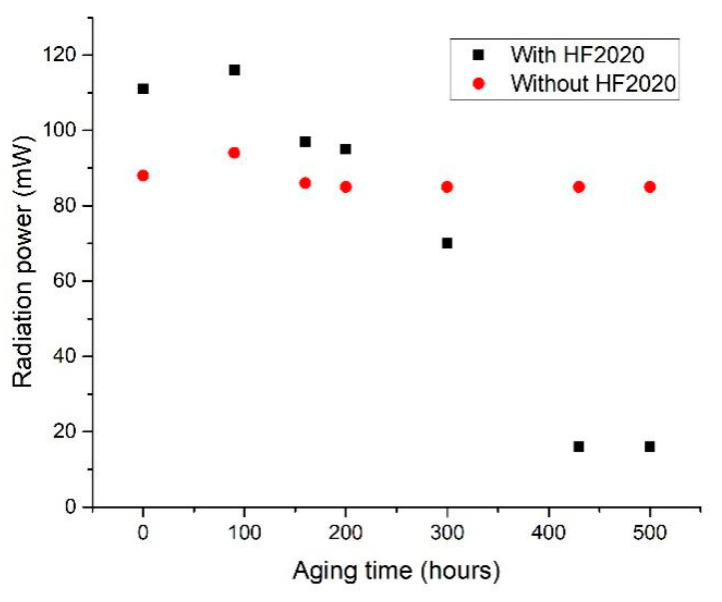
Device aging test for the UVC LED chip with and without HF2020 encapsulation.

**Figure 6:**
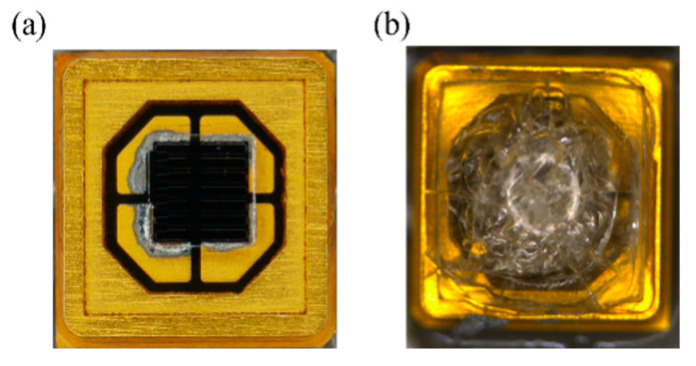
The morphology of UVC LED (**a**) before the aging test, and (**b**) after 500 h of aging time.

Figure 5 shows the radiant power characterization results of the aging test. The encapsulation material, HF2020, was solidified in the first ten hours after the aging test began. In the first 200 h, the encapsulated LED chip showed higher radiant power than the bare UVC LED chip. However, cracks in the solidified HF2020 were generated and propagated after 200 h, and the radiant power of UVC LED with HF2020 decreased dramatically after 200 h. The encapsulation material was full of cracks at the end of the aging test, as shown in Figure 6. Cracks scatter the light emitted from the LED chip and the encapsulation material becomes opaque. Cracks led to a dramatic drop down of the radiant power. The increase in the Si-O-Si bond became oversaturated after surpassing the saturation threshold indicated in Figure 4. The oversaturation of the Si-O-Si bonds may lead to an enhanced degree of crosslinking, which consequently results in increased internal stress and brittleness in HF2020. Cracks were possibly caused by thermal stress and UV aging effects [15], and this element can be improved by future development of composition optimization. Due to its curing property and high radiant power in the first 200 h, bio-compatibility, high 265 nm transmittance, and relatively long lifetime, HF2020 has the potential to become an excellent encapsulation material for implantable UVC LED applications.

## 4. Conclusions

To develop new encapsulation materials for UVC LED packaging, a series of material aging experiments were performed on CF1040, HF2020, and MF2020-1000 under UVC radiation for 1000 h. HF2020 demonstrated excellent transmittance performance during the aging experiments, resulting in the highest radiant power among the tested materials. The experimental results indicate an increase in transmittance of HF2020 before 500 h of aging, suggesting a curing process during this period. The solidification speed of HF2020, attributed to the photolysis of Si-H bonds leading to the formation of Si-O-Si, is the fastest among the tested materials.

To assess the practical effects of HF2020 encapsulation, a device aging test was conducted. Testing of 265 nm UVC LEDs, both with and without HF2020 encapsulation, revealed that the encapsulated LEDs achieved a significant maximum increase of 27% in radiant power compared to their unencapsulated counterparts. Furthermore, these encapsulated LEDs maintained higher radiant power levels during the initial 200 h of operation. This enhancement in performance underscores their potential application in photodynamic therapy. By activating photosensitizers through increased UVC exposure, these LEDs promote the rapid generation of reactive oxygen species, which can effectively destroy cancer cells in a brief period.

## 5. Challenges and Future Prospects

This study highlights the promising potential of silicone oil as a material for encapsulating UVC LEDs. However, with a lifespan of only 200 h, it falls short of the requirements for commercial use. To improve the longevity of silicone oil in this application, collaborative efforts between industry and academia are essential for developing more durable silicone oil formulations tailored to UVC LED encapsulation. Furthermore, considering the current lifespan limitations and the biocompatibility of silicone oil, UVC LED devices encapsulated with this material could be utilized as implanted UVC light sources to target and eliminate cancer cells. This application could significantly expand the options available for photodynamic therapy in cancer treatment.

## Figures and Tables

**Table 1:** Viscosity result of different silicone oils.

Viscosity (cP)	Aging Time (Hour)						
	0	150	250	350	500	750	1000
CF1040	1.5	1.8	2	1.5	1.8	1.8	1.8
MF2010-1000	900	1290	2370	5550	6300	colloidal solid	colloidal solid
HF2020	20	146	solid	solid	solid	solid	solid

**Table 2:** Relationship between the UVC LED radiant power and HF2020 volume.

	Number	1	2	3	4
Item					
Volume Dispensed (HF2020)		2.5 μL	3.0 μL	3.5 μL	4.0 μL (maximum volume dispensed)
Morphology		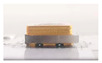	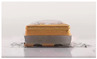	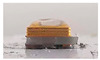	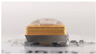
Radiant Power		94 mW	102 mW	112 mW	125 mW
Normalized Radiant Power		100%	109%	119%	133%

**Table 3:** Comparison of the radiant power of the bare UVC LED chip and the UVC LED chip encapsulated with 4.0 μL of HF2020.

Comparison of the Radiant Power of the Bare UVC LED Chip and the UVC LED Chip Encapsulated with 4.0 μL of HF2020	Bare UVC LED Chip	UVC LED Chip Encapsulated with 4.0 μL of HF2020
Radiant power (mW)	98	125
Normalized radiant power	100%	127%

## Data Availability

The original contributions presented in this study are included in the article. Further inquiries can be directed to the corresponding author.
